# Single-Particle Tracking of AMPA Receptor-Containing Vesicles

**DOI:** 10.21769/BioProtoc.5325

**Published:** 2025-06-05

**Authors:** Victor C. Wong, Deepika Walpita, Zhe J. Liu, Erin K. O’Shea

**Affiliations:** Janelia Research Campus, Howard Hughes Medical Institute, Ashburn, VA, USA

**Keywords:** AMPA receptor-containing vesicles (AMPAR^+^ vesicles), AMPAR GluA1 subunit (GluA1), HaloTag (HT), Homology-independent targeted integration (HITI), Janelia Fluor (JF) dyes, Live-cell timelapse imaging, Single-particle tracking (SPT), Cultured rat hippocampal neurons

## Abstract

AMPA-type receptors are transported large distances to support synaptic plasticity at distal dendritic locations. Studying the motion of AMPA receptor^+^ vesicles can improve our understanding of the mechanisms that underlie learning and memory. Nevertheless, technical challenges that prevent the visualization of AMPA receptor^+^ vesicles limit our ability to study how these vesicles are trafficked. Existing methods rely on the overexpression of fluorescent protein-tagged AMPA receptors from plasmids, resulting in a saturated signal that obscures vesicles. Photobleaching must be applied to detect individual AMPA receptor^+^ vesicles, which may eliminate important vesicle populations from analysis. Here, we present a protocol to study AMPA receptor^+^ vesicles that addresses these challenges by 1) tagging AMPA receptors expressed from native loci with HaloTag and 2) employing a block-and-chase strategy with Janelia Fluor-conjugated HaloTag ligand to achieve sparse AMPA receptor labeling that obviates the need for photobleaching. After timelapse imaging is performed, AMPA receptor^+^ vesicles can be identified during image analysis, and their motion can be characterized using a single-particle tracking pipeline.

Key features

• Track and characterize the motion of AMPAR GluA1^+^ vesicles in cultured rat hippocampal neurons.

• GluA1 tagged with HaloTag (GluA1-HT) is expressed from native *Gria1* loci to avoid overexpression.

• Sparse GluA1-HT labeling densities can be achieved without photobleaching via a block-and-chase strategy that utilizes Janelia Fluor (JF) dyes conjugated to HaloTag ligand (HTL).

• GluA1-HT^+^ vesicles are identified during image analysis, and their motion is characterized using single-particle tracking (SPT) and hidden Markov modeling with Bayesian model selection (HMM-Bayes).

## Graphical overview


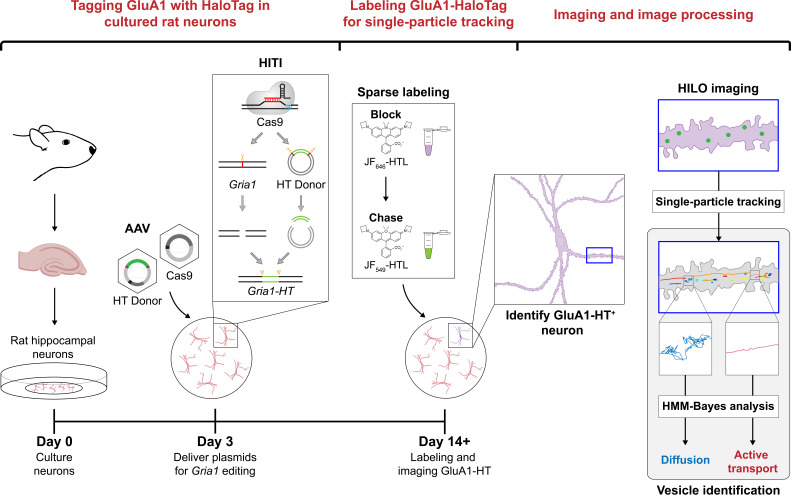


**Block-and-chase labeling of GluA1-HaloTag expressed from the genomic loci of *Gria1* facilitates single-particle tracking of GluA1-HaloTag^+^ vesicles**

## Background

α-amino-3-hydroxy-5-methyl-4-isoxazolepropionic acid receptors (AMPARs) are ionotropic glutamate receptors that are rapidly inserted into and removed from synapses during synaptic plasticity [1]. Changes in the concentration and conductance of AMPARs in synapses during neuronal activity are thought to form the basis of functional plasticity [2]. Consequently, elucidating how AMPARs are trafficked to synapses is key to understanding the molecular underpinnings of learning and memory.

The majority of AMPARs are synthesized in the cell body, mature as they are trafficked through the secretory pathway, and eventually undergo exocytosis onto the neuronal surface [3]. AMPARs diffuse through the neuronal membrane but accumulate in synapses due to selective retention [4–8]. During potentiation and other forms of plasticity, the structure and protein composition of synapses change to enhance the capture of AMPARs [9–13]. While the diffusional properties of AMPARs on the neuronal surface have been studied extensively [4–10], how AMPAR^+^ vesicles are transported to specific distal dendritic locations, and whether the vesicular transport of AMPARs supports plasticity at these locations, is not fully understood.

The motion of AMPAR^+^ vesicles is difficult to study, largely due to technical obstacles that prevent the observation of vesicles in live neurons [10]. To study the motion of AMPAR^+^ vesicles, AMPARs must be sparsely labeled so that the motion of single vesicles can be unambiguously tracked. Expressing AMPARs tagged with fluorescent proteins (e.g., GFP) from plasmids results in a saturated AMPAR signal [14], preventing the identification of single AMPAR^+^ vesicles. Consequently, sparse labeling is achieved through photobleaching sections of neurites and then tracking AMPAR^+^ vesicles as they are transported through the photobleached region [15]. Using this technique, interesting motion characteristics have been described. For example, AMPAR^+^ vesicles pause in response to the influx of calcium during neuronal activity [15]. Nevertheless, the overexpression of AMPARs suffers from drawbacks that may limit the physiological relevance of findings. First, overexpressing one AMPAR subunit may lead to the excess formation of AMPAR homomers (e.g., non-physiological cellular concentrations of calcium-permeable GluA1 homomers) [12]. Overexpression may also lead to aberrant trafficking, such as retention in the ER or excess accumulation in synapses [16]. Lastly, because photobleaching is required to achieve sparse labeling, this strategy may bias the detection of AMPAR^+^ vesicles that are actively transported through the photobleached region and undercount vesicles that have slow or diffusive motion.

Here, we present a protocol for tracking AMPAR^+^ vesicles that does not rely on the overexpression of AMPAR subunits or photobleaching. We use homology-independent targeted integration (HITI) [17] to insert HaloTag (HT) [18] into the genomic loci of *Gria1*, the gene encoding the AMPAR GluA1 subunit. When expressed, endogenous GluA1 contains HT fused to its extracellular amino-terminal domain (ATD) and can thus be labeled with dyes conjugated to the HaloTag ligand (HTL). We subsequently employ a block-and-chase protocol to sparsely label GluA1-HT with Janelia Fluor (JF) dyes [19] that have been conjugated to HTL, allowing us to easily and unambiguously identify single GluA1-HaloTag (GluA1-HT) particles. We then track these particles using a multiple target tracing (MTT) algorithm for single-particle tracking (SPT) [20] and characterize their motion using hidden Markov modeling with Bayesian model selection (HMM-Bayes) [21]. By analyzing the motion and bleaching rate of GluA1-HT particles, we can distinguish GluA1-HT^+^ vesicles from GluA1-HT on the surface of the cell. This technique can be used to study the motion of particles in unstimulated conditions or during neuronal activity (e.g., activity induced by glutamate uncaging). Although we have used this method to study the motion of GluA1^+^ vesicles, we believe this protocol can be applied to other important proteins if appropriate HaloTag insertion sites can be identified and validated.

## Materials and reagents

**Biological materials**

1. Sprague-Dawley rats, CD (Sprague-Dawley postnatal pups P0, M, and F; Charles River Laboratories, strain code: 001)

2. PX551 (Cas9) plasmid (Addgene, catalog number: 60957)

3. px552-sg-gria1-HT (donor) plasmid (Addgene, catalog number: 187652)

4. rh10-PX551 AAV (Janelia Research Campus Viral Services)

5. rAAV2-px552-sg-gria1-HT AAV (Janelia Research Campus Viral Services)

**Reagents**

1. Poly-D-lysine (PDL) hydrobromide, MW >300,000 (Sigma-Aldrich, catalog number: P7405)

2. Hanks’ balanced salt solution (HBSS), 1× (Thermo Fisher Scientific, catalog number: 24020117)

3. HEPES solution, 1 M (Thermo Fisher Scientific, catalog number: 15630106)

4. Papain (Worthington Biochemical Corporation, catalog number: PAP2 LK003178)

5. Benzonase nuclease, 25 KU (Millipore Sigma, catalog number: E1014)

6. Minimal essential media (MEM) (Thermo Fisher Scientific, catalog number: 11090081)

7. Fetal bovine serum (FBS) (Thermo Fisher Scientific, catalog number: 10437028)

8. D-glucose (Millipore Sigma, catalog number: G7021)

9. Sodium bicarbonate (NaHCO_3_) (Millipore Sigma, catalog number: S8875)

10. Insulin solution, 10 mg/mL (Millipore Sigma, catalog number: I0516)

11. Transferrin (Millipore Sigma, catalog number: 616420)

12. L-glutamine solution, 200 mM (Thermo Fisher Scientific, catalog number: 25030081)

13. Penicillin-streptomycin (Pen-Strep), 10,000 units/mL (100×) (Thermo Fisher Scientific, catalog number: 15140122)

14. NbActiv4 minus phenol red (Transnetyx, catalog number: NB4PR500)

15. JF_646_-HaloTag ligand (JF_646_-HTL) (Janelia Research Campus Materials)

16. JF_549_-HaloTag ligand (JF_549_-HTL) (Janelia Research Campus Materials)

17. Dimethyl sulfoxide (DMSO), anhydrous (Thermo Fisher Scientific, catalog number: D12345)

18. Cycloheximide solution (CHX), 100 mg/mL (Sigma-Aldrich, catalog number: C4859)

19. Sodium chloride (NaCl) (Millipore Sigma, catalog number: S5886)

20. Calcium chloride solution (CaCl_2_), 1 M (Millipore Sigma, catalog number: 21115)

21. Magnesium chloride solution (MgCl_2_), 1 M (Thermo Fisher Scientific, catalog number: AM9530G)

22. Potassium chloride (KCl) (Millipore Sigma, catalog number: P5405)

23. HEPES (Millipore Sigma, catalog number: H4034)

24. Sterile Milli-Q H_2_O

**Solutions**

1. 0.5 mg/mL Poly-D-lysine stock solution (see Recipes)

2. Dissection buffer (see Recipes)

3. Papain dissociation solution (see Recipes)

4. Plating media (see Recipes)

5. Complete P3 primary cell Nucleofector^®^ solution (see Recipes)

6. 200 μM JF_646_-HTL and JF_549_-HTL stock solutions (see Recipes)

7. JF_646_-HTL blocking solution (see Recipes)

8. JF_549_-HTL labeling solution (see Recipes)

9. Imaging buffer (see Recipes)

**Recipes**

**1. 0.5 mg/mL poly-D-lysine stock solution (10 mL)**

**Table:** 

Reagent	Final concentration	Quantity or Volume
PDL hydrobromide	0.5 mg/mL	5 mg
H_2_O	n/a	10 mL

*Note: Make 1 mL aliquots and store at -20 °C.*

**2. Dissection buffer (500 mL)**

**Table:** 

Reagent	Final concentration	Quantity or Volume
HBSS	n/a	490 mL
1 M HEPES	10 mM	5 mL
100× pen-strep	1×	5 mL

*Note: Store at 4 °C.*

**3. Papain dissociation solution (5 mL)**

**Table:** 

Reagent	Final concentration	Quantity or Volume
Papain	n/a	1× glass vial
Dissection buffer	n/a	5 mL
25 KU Benzonase nuclease	n/a	0.75 μL

*Notes:*

*1. Add 5 mL of dissection buffer into the vial containing papain and gently swirl until the powder has dissolved completely. Transfer the solution into a 15 mL conical tube and add 0.75 μL of Benzonase nuclease to the solution.*

*2. Make fresh papain dissociation solution right before dissecting the animal.*

*3. Keep the complete solution on ice until use.*

**4. Plating media (500 mL)**

**Table:** 

Reagent	Final concentration	Quantity or Volume
MEM	n/a	440 mL
FBS	10%	50 mL
D-glucose	5 mg/mL	2.5 g
NaHCO_3_	0.2 mg/mL	0.1 g
10 mg/mL insulin	0.025 mg/mL	1.25 mL
Transferrin	0.1 mg/mL	50 mg
200 mM L-glutamine	2 mM	5 mL
100× pen-strep	1×	5 mL

*Notes:*

*1. To dissolve transferrin, add 2 mL of incomplete MEM (no supplements added) to the 100 mg transferrin vial. Place the vial into a 37 °C water bath until the transferrin has dissolved into a clear, light brown solution. Take 1 mL (50 mg) of the solution and add to the MEM bottle.*

*2. To dissolve D-glucose, add 2.5 g of D-glucose to the bottle of MEM and place in a 37 °C water bath for 30 min. Gently swirl the bottle every 10 min. Alternatively, D-glucose can be added to the bottle of MEM and allowed to dissolve at 4 °C overnight.*

*3. Do not repeatedly freeze-thaw L-glutamine. Make 5 mL aliquots and store at -20 °C. If thawed L-glutamine has precipitated, warm the solution for 1 min in a 37 °C water bath and mix. Do not leave L-glutamine in a water bath for longer than 1 min.*

*4. Mix all supplements added to the bottle of MEM and sterile filter through a vacuum-driven 0.22 μm CA membrane.*

*5. Store at 4 °C.*

**5. Complete P3 primary cell Nucleofector^®^ solution (22 μL)**

**Table:** 

Reagent	Final concentration	Quantity or Volume
P3 Nucleofector^®^ solution	n/a	18 μL
Supplement 1	n/a	4 μL

*Notes:*

*1. Proprietary solutions are part of the P3 primary cell 96-well Nucleofector^®^ kit (Lonza).*

*2. Volumes described here are sufficient for one sample (i.e., one nucleofection).*

*3. Store according to the manufacturer’s directions.*

**6. 200 μM JF_646_-HTL and JF_549_-HTL stock solutions (500 μL)**

**Table:** 

Reagent	Final concentration	Quantity or Volume
JF_646_-HTL or JF_549_-HTL	200 μM	100 nmol
DMSO	n/a	500 μL

*Note: Make 50 μL aliquots and store at -20 °C. Avoid exposure to light.*

**7. JF_646_-HTL blocking solution (2 mL)**

**Table:** 

Reagent	Final concentration	Quantity or Volume
20 μM JF_646_-HTL	20 nM	2 μL
10 mg/mL CHX	50 nM	2.8 μL
NbActiv4	n/a	2 mL

*Notes:*

*1. Dilute the 200 μM JF_646_-HTL and 100 mg/mL CHX stock solutions 1:10 in NbActiv4 before making the blocking solution.*

*2. Make the solution immediately before use in prewarmed NbActiv4 (37 °C).*

*3. Avoid exposure to light.*

**8. JF_549_-HTL labeling solution (2 mL)**

**Table:** 

Reagent	Final concentration	Quantity or Volume
20 μM JF_549_-HTL	10 nM	1 μL
NbActiv4	n/a	2 mL

*Notes:*

*1. Dilute the 200 μM JF_549_-HTL stock solution 1:10 in NbActiv4 before making the labeling solution.*

*2. Make the solution immediately before use in prewarmed NbActiv4 (37 °C).*

*3. Avoid exposure to light.*

**9. Imaging buffer (500 mL)**

**Table:** 

Reagent	Final concentration	Quantity or Volume
NaCl	150 mM	4.383 g
1 M CaCl_2_	2 mM	1 mL
1 M MgCl_2_	2 mM	1 mL
KCl	5 mM	0.185 g
HEPES	10 mM	1.192 g
D-glucose	30 mM	2.702 g
H_2_O	n/a	Final volume to 500 mL

*Note: Adjust to pH 7.4 with NaOH. Store at 4 °C.*

**Laboratory supplies**

1. 40 μm Falcon cell strainers (Thermo Fisher Scientific, catalog number: 08-771-1)

2. P3 primary cell 96-well Nucleofector^®^ kit (Lonza, catalog number: V4SP-3096)

3. 1.5 mL Eppendorf tubes (Thermo Fisher Scientific, catalog number: 05-402-27)

4. 15 mL Falcon conical tubes (Thermo Fisher Scientific, catalog number: 14-959-53)

5. 50 mL Falcon conical tubes (Thermo Fisher Scientific, catalog number: 14-432-22)

6. Glass-bottom dishes, No 1.5 coverglass (MatTek, catalog number: P35G-1.5-14-C)

7. ART barrier pipette tips, P10–P1000 (Thermo Fisher Scientific, catalog numbers: 2139, 2149, 2069, 2179)

8. 500 mL bottle top vacuum filter, 0.22 μm pore cellulose acetate (CA) membrane (Corning, catalog number: 430521)

## Equipment

1. 4D-Nucleofector^®^ 96-well unit (Lonza Bioscience, catalog number: AAF-1003S)

2. 4D-Nucleofector^®^ core unit (Lonza Bioscience, catalog number: AAF-1003B)

3. Vi-CELL BLU (Thermo Fisher Scientific, catalog number: C19196)

4. Centrifuge 5810R (Eppendorf, catalog number: 022625501)

5. HERAcell VIOS 250i CO_2_ incubator (Thermo Fisher Scientific, catalog number: 50162755)

6. Nikon Eclipse TiE inverted microscope (Nikon, product number: MEA53100)

*Note: Key components are listed below.*

a. Ti-ND6-PFS perfect focus (Nikon, product number: MEP59391)

b. CFI60 Apochromat TIRF 100× oil immersion objective lens N.A. 1.49 (Nikon, product number: MRD01991)

c. TIRF illuminator and components (Nikon, custom order)

d. LUNV laser unit with lines: 405 nm/488 nm/561 nm/647 nm (Nikon, product number: NIIMHF47060)

e. iXon DU897 ultra-electron multiplying charge-coupled device (EMCCD) cameras (x3) (Andor, product number: 77026047)

f. TriCam LS (Nikon, product number: 77072033)

g. Stage top TIZW Series Neco incubation system (Tokai HIT, product number: 77025086)

## Software and datasets

1. NIS-Elements C imaging software (Nikon, version 5.21.00)

2. MATLAB (MathWorks, version 2015B)

3. SLIMfast.m (single-particle tracking pipeline, DOI: 10.5281/zenodo.14901152)

4. DiffusionSingle.m (single-particle tracking pipeline, DOI: 10.5281/zenodo.14901152)

5. TrackValidator.m (single-particle tracking pipeline, DOI: 10.5281/zenodo.14901152)

6. HMM_Batch.m (single-particle tracking pipeline, DOI: 10.5281/zenodo.14901152)

## Procedure

**A. Preparation of poly-D-lysine-coated MatTek glass-bottom dish**

*Note: Prepare solutions and coat MatTek dishes with PDL inside a biosafety cabinet. Use sterile technique to avoid contamination.*

1. Dilute 0.5 mg/mL PDL stock solution 1:10 to a working concentration of 0.05 mg/mL in sterile Milli-Q H_2_O.

2. Add 200 μL of 0.05 mg/mL PDL to the center of the glass coverslip of a MatTek glass-bottom dish.

3. Using a pipette tip, gently spread the PDL to cover the edges of the coverslip.

4. Incubate the MatTek dish with PDL for 2 h at room temperature inside a biosafety cabinet.

5. Aspirate PDL from the glass coverslip of the MatTek dish and wash the dish three times using 500 μL of sterile H_2_O for 5 min each wash.

**B. Preparation of primary rat hippocampal neuron culture**

*Notes:*

*1. All neuronal culture steps should be performed inside a biosafety cabinet. Use sterile technique to avoid contamination.*

*2. Handle tissues and cells with care. Do not pipette with excessive force.*

*3. Plating media and NbActiv4 should be prewarmed to 37 °C prior to use.*

*4. Keep fresh papain dissociation solution on ice until 5 min before use.*

1. Hippocampal neurons were prepared from P0 Sprague-Dawley rat pups (Charles River) according to institutional guidelines and protocol (IACUC protocol number 24-0258, protocol title: “Procurement of animal tissue for research applications”). Protocols for isolating the hippocampus from P0 rat pups have been described in detail elsewhere [22].

*Note: We collect hippocampi from 2–4 rat pups and perform dissociation on all hippocampi in a batch.*

2. Once hippocampi are excised, cut each hippocampus into 3–4 smaller pieces using a sterile razor blade.

3. Remove papain dissociation solution from ice and allow it to sit at room temperature for 5 min.

4. Transfer dissected hippocampal tissue pieces to a 15 mL conical tube and briefly allow tissue pieces to settle at the bottom of the tube.

*Note: Create a wide-bore P1000 tip by cutting the end of a P1000 tip with sterile scissors. This wide-bore tip can be used to transfer the tissue.*

5. Carefully remove any solution from the conical tube using a P1000 pipette.

6. Add 1 mL of papain dissociation solution for approximately 6–8 pieces of hippocampi from two rat pups.

*Note: Scale up the volume of papain dissociation solution accordingly.*

7. Place the tube with the hippocampal tissue into a 37 °C water bath for 30 min for digestion. Gently swirl the tube every 5–7 min to ensure the enzyme digests all surfaces of the tissue.

8. After 30 min of digestion, remove the tube from the water bath and briefly allow tissue pieces to settle at the bottom of the conical tube.

9. Carefully remove papain dissociation solution using a P1000 pipette.

10. Add 1 mL of prewarmed plating media to the tube.

11. Using a P1000 pipette, gently triturate the digested tissue 3–4 times.

12. Add 5 mL of prewarmed plating media to the tube and allow large, undissociated tissue pieces to settle to the bottom of the tube for >1 min. Single cells, small cell clumps, and some tissue debris will remain in the supernatant.

13. Prepare a 50 mL conical tube with a 40 μm cell stainer and add 1 mL of prewarmed plating media to wet the membrane to allow cells to smoothly pass through the filter.

14. Using the P1000 pipette, gently transfer the cell supernatant and pass cells through the 40 μm cell stainer.

15. Repeat steps B10–14 on the remaining hippocampal tissue inside the 15 mL conical tube.

16. After all hippocampal tissue has been dissociated and filtered, centrifuge the cell suspension at 100× *g* for 8 min.

17. Remove supernatant containing cell debris from the cell pellet, add 1 mL of prewarmed plating media, and gently dislodge the cell pellet from the tube.

18. Add an additional 4 mL of prewarmed plating media.

19. Determine the cell density and viability of the cell suspension. We only use cell preparations if viability is greater than 90%.

*Note: We use a Vi-CELL BLU cell viability analyzer according to the manufacturer’s instructions. When determining the viability and density of cells, inspect the images taken by the cell counter to ensure that the suspension consists mostly of single cells and not cell clumps or debris.*

20. Plate 50,000 cells at the center of each PDL-coated MatTek dish to be used.

21. Add an equivalent volume of prewarmed NbActiv4 to the cells and gently rock the MatTek dish to spread the cells onto the PDL-coated coverslip.

22. Allow cells to adhere onto PDL for at least 3 h in the incubator at 5% CO_2_ and 37 °C.

23. Gently add 2 mL of prewarmed NbActiv4 into the dish and return the dish to the incubator.

24. Replace 1 mL of NbActiv4 in the dish with fresh, prewarmed NbActiv4 every 7 days until neurons are ready for use (after DIV10).

**C. Adeno-associated virus transduction of cultured rat neurons with plasmids to insert HaloTag into *Gria1***

*Notes:*

**Figure 1.**
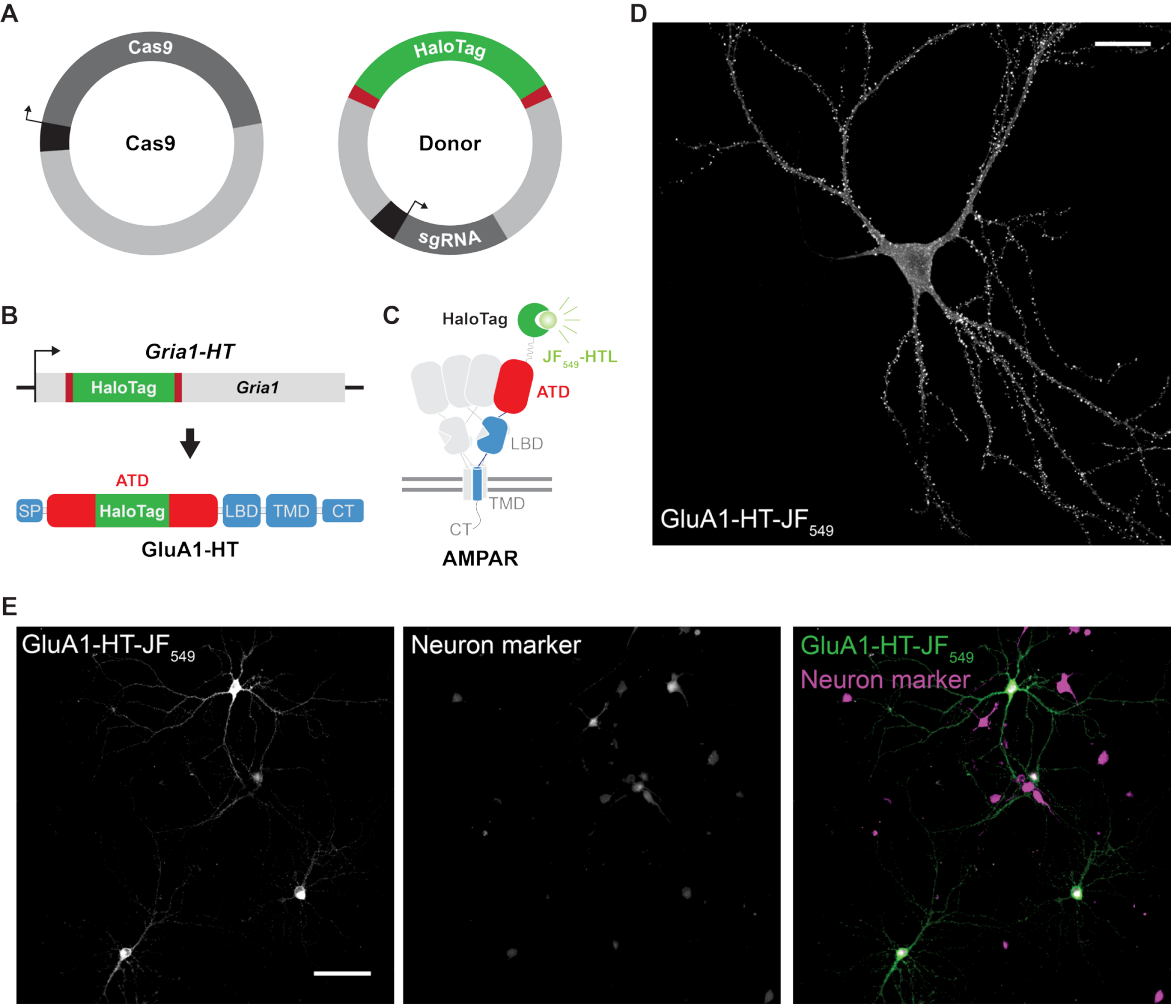
Homology-independent targeted integration can be used to insert HaloTag into *Gria1* in cultured rat hippocampal neurons. (A) Constructs used to insert HaloTag into the genomic loci of *Gria1* using homology-independent targeted integration (HITI). The donor construct (Donor) contains the DNA sequence coding for HaloTag (HaloTag) and the single guide RNA that will target Cas9 to *Gria1* (sgRNA). Importantly, the HaloTag coding sequence is flanked by *Gria1* sequences that will be targeted and cut by Cas9 (red bars). Cas9 is expressed from a second plasmid (Cas9). Black bars represent promoters driving the expression of Cas9 and the sgRNA. (B) Cas9 creates double-strand breaks in the genomic loci of *Gria1* and releases HaloTag from the Donor. HaloTag can be inserted into *Gria1* by non-homologous end joining (NHEJ). When expressed from the edited copy of *Gria1 (Gria1-HT*), GluA1 will contain HaloTag inserted into its amino-terminal domain (ATD; GluA1-HT). SP, signal peptide; LBD, ligand-binding domain; TMD, transmembrane domain; CT, C-terminal tail. (C) Diagram of AMPAR containing a GluA1 subunit (blue) with HaloTag (green) inserted into the ATD (red). AMPARs are tetramers that can contain zero to four GluA1 subunits. As HaloTag is inserted into the ATD, it will sit on the extracellular side of the receptor. HaloTag can be labeled with fluorescent dyes that are conjugated to HaloTag ligand (HTL), such as JF_549_-HTL. (D) Representative confocal image of a cultured rat hippocampal neuron expressing GluA1 tagged with HaloTag and labeled with JF_549_-HTL (GluA1-HT-JF_549_). Scale bar, 25 μm. (E) Representative widefield images of edited neurons expressing GluA1-HT labeled with JF_549_-HTL (GluA1-HT-JF_549_) and a fluorescent neuron marker (in this case, miRFP670 driven by a synapsin promoter). Scale bar, 100 μm.

*1. HaloTag is inserted into GluA1 using homology-independent targeted integration (HITI). This process involves transfecting or transducing a neuron with two plasmids: 1) a Cas9 expression construct and 2) a donor plasmid that contains the coding sequence for HaloTag (Figure 1A). Cas9 will create a double-strand break in* Gria1 *and release the HaloTag coding sequence from the donor plasmid, after which HaloTag can be inserted into* Gria1 *by non-homologous end joining (NHEJ) (Figure 1B, top). When expressed, GluA1 will be tagged with HaloTag in its amino-terminal domain (ATD) (Figure 1B, bottom). Mature receptors can be labeled with HaloTag ligand that has been conjugated with fluorescent dyes or other molecules (Figure 1C).*

*2. We recommend using adeno-associated virus (AAV) to deliver the Cas9 (PX551) and HaloTag donor (px552-sg-gria1-HT) plasmids into neurons. AAV infection results in the highest knock-in efficiency (~2.6% of cells transduced with both plasmids contain a HaloTag knock-in [23]) and has significantly less toxicity when compared to nucleofection. AAVs can be pseudotyped with AAV2/9 and rh10 capsids for efficient transduction. We use AAV packaged by the Janelia Research Campus Viral Services. For the protocol, please see [23].*

*3. Neurons can be infected with AAV between 3 and 14 days in vitro (DIV 3–14). Infecting the neurons at DIV 3 results in the highest rate of infection, which decreases as neurons age in vitro.*

*4. The addition of virus to neuronal cultures should be performed inside a biosafety cabinet. Use sterile technique to avoid contamination.*

*5. NbActiv4 should be prewarmed to 37 °C prior to use.*

1. Mix 1 × 10^10^ genomic copies of rh10-PX551 (Cas9) and 1 × 10^10^ genomic copies of rAAV2-px552-sg-gria1-HT (donor) into 50 μL of prewarmed NbActiv4.

2. Add the entire volume of virus in NbActiv4 directly into the media of the culture dish and gently swirl to mix.

3. Return the culture dish to the incubator at 5% CO_2_ and 37 °C until cells are ready for use. HaloTag^+^ cells can sometimes be identified as early as 4 days after infection (i.e., DIV 7), but it may be necessary to wait 7 days after infection (DIV 10).

**D. Nucleofection (alternative to AAV infection)**

*Notes:*

*1. If AAVs of PX551 and px552-sg-gria1-HT are not available, plasmids can be used for transfection via nucleofection when neurons are extracted and cultured on P0. However, nucleofection-based HITI has relatively low knock-in efficiency (~1.7% of cells transfected with both plasmids will contain a HaloTag knock-in [23]) and high toxicity when compared to AAV-based HITI.*

*2. All neuronal culture steps should be performed inside a biosafety cabinet. Plasmids and solutions should also be prepared inside a biosafety cabinet. Do not remove the lid of the Nucleocuvette^®^ plate outside of the biosafety cabinet to avoid contamination. Use sterile technique to avoid contamination.*

*3. Handle cells with care, especially after nucleofection. Avoid applying excessive shearing force during pipetting.*

*4. Warm complete P3 Nucleofector^®^ solution to room temperature before use.*

*5. Prewarm plating media and NbActiv4 to 37 °C prior to use.*

*6. The number of cells and volumes of solutions described below are sufficient for one sample (one transfection). Scale up accordingly for the desired number of transfections.*

1. Prior to dissection, prepare a 1.5 mL Eppendorf tube with the donor and Cas9 expression plasmids by pipetting 0.5 μg of PX551 and 0.5 μg of px552-sg-gria1-HT in a 1.5 mL Eppendorf tube.

*Note: Dilute stock concentrations of plasmids to 0.5 μg/μL and then use 1 μL of each.*

2. After performing steps B1–19, calculate and transfer 500,000 cells into a 1.5 mL Eppendorf tube for transfection.

3. Gently pellet cells at 90× *g* for 5 min at room temperature in a centrifuge.

4. Carefully remove media from the cell pellet and resuspend cells in complete P3 Nucleofector^®^ solution (P3 Nucleofector^®^ solution plus supplement 1). Use 20 μL of complete P3 Nucleofector^®^ solution for every 500,000 cells.

5. Transfer 20 μL of cells in complete P3 Nucleofector^®^ solution to the 1.5 mL Eppendorf tube containing 0.5 μg of PX551 and 0.5 μg of px552-sg-gria1-HT.

6. Transfer the entire volume of cells into one well of a 96-well Nucleocuvette^®^ plate and gently tap the plate to ensure that the cell suspension has settled at the bottom of the well without bubbles.

7. Load the plate onto 4D-Nucleofector^®^ and transfect cells using code CU-110.

8. Add 80 μL of plating media into the well and allow cells to recover by placing the plate into the incubator at 5% CO_2_ and 37 °C for 5 min.

9. Transfer the entire volume inside the well (~100 μL) to 500 μL of prewarmed plating media in a 1.5 mL Eppendorf tube.

10. Plate 50,000 cells at the center of the PDL-coated MatTek dish.

11. Add an equivalent volume of prewarmed NbActiv4 to help spread the cells onto the PDL coating.

12. Allow cells to adhere onto PDL for at least 3 h in the incubator at 5% CO_2_ and 37 °C.

13. Gently add 2 mL of prewarmed NbActiv4 to the dish and return the dish to the incubator.

14. Replace 1 mL of NbActiv4 in the dish with fresh, prewarmed NbActiv4 every 7 days until neurons are ready for use (after DIV10).

**E. Labeling cultured neurons that express GluA1-HaloTag with HaloTag ligand conjugated with Janelia Fluor dyes**

*Notes:*

*1. At DIV 10 (7 days after AAV infection or 10 days after nucleofection), neurons can be labeled with JF_646_- or JF_549_-HTL to examine whether* Gria1 *was edited with HaloTag.*

*2. Prewarm NbActiv4 and imaging buffer to 37 °C prior to use.*

*3. Perform labeling and wash steps inside a biosafety cabinet and use sterile technique.*

1. Prepare 20 nM JF_646_-HTL or 10 nM JF_549_-HTL in 2 mL of prewarmed NbActiv4.

2. Aspirate most of the NbActiv4 from the neuronal culture dish (save a small volume of NbActiv4 covering the cells on the coverslip) and replace with NbActiv4 containing JF_646_-HTL or JF_549_-HTL.

3. Return the dish to the incubator for 30 min.

4. Aspirate NbActiv4 with JF_646_-HTL or JF_549_-HTL and replace with 2 mL of prewarmed imaging buffer. Repeat this wash three more times to remove unbound dye from the culture dish.

5. View cells under a microscope with a 10× or 20× objective. Once a positive cell has been identified, switch to a higher magnification objective to view the neuron in detail. JF_646_ can be excited with a 640 nm laser line or LED light with filters appropriate for Cy5. JF_549_ can be excited with a 561 nm laser line or LED light with filters appropriate for TRITC. Edited neurons should have GluA1-HT labeling in the cell body and dendrites, and especially strong labeling in the dendritic spines where GluA1 accumulates (Figure 1D).

*Note: A neuronal transfection marker may be used to confirm that cells expressing GluA1-HT are neurons and to estimate the transfection/transduction efficiency (Figure 1E). For example, we use the fluorescent protein miRFP670 driven by the neuron-specific synapsin promoter. See* ***Troubleshooting #4*** *for more details.*

**F. Sparse labeling de novo synthesized GluA1-HaloTag using block-and-chase labeling protocol**

*Notes:*

**Figure 2.**
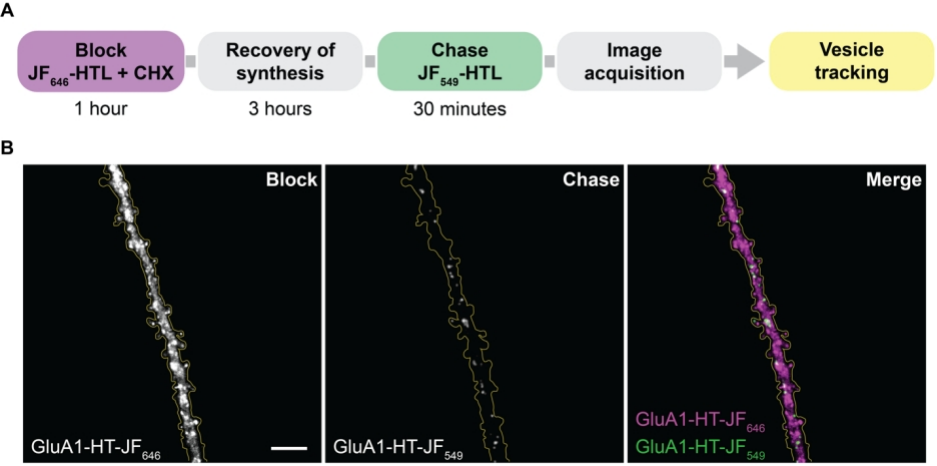
Block-and-chase protocol to sparsely label GluA1-HaloTag. (A) Workflow to sparsely label GluA1-HT with Janelia Fluor dyes. (B) Representative widefield image of a dendrite from a cultured rat hippocampal neuron expressing GluA1-HT first labeled with JF_646_-HTL (block; GluA1-HT-JF_646_) and then JF_549_-HTL (chase; GluA1-HT-JF_549_). Scale bar, 10 μm.

*1. Workflow overview is depicted in* ***Figure 2A***.

*2. At DIV 14, cultured neurons are sufficiently developed to easily identify healthy, spiny dendrites. Consequently, labeling and imaging are best performed between DIV 14 and 21.*

*3. Prewarm NbActiv4 and imaging buffer to 37 °C prior to use.*

*4. Perform labeling and wash steps inside a biosafety cabinet and use sterile technique.*

*5. CHX is added to the blocking solution to inhibit the synthesis of GluA1-HT during the blocking step. After all existing protein is blocked by JF_646_-HTL, protein synthesis is allowed to recover by removing CHX. During the chase step, only newly synthesized GluA1-HT will be labeled with JF_549_-HTL. Because the newly synthesized GluA1-HT makes up only a small fraction of total GluA1-HT, the JF_549_ signal will be sparse enough for single-particle tracking.*

1. Prepare 2 mL of JF_646_-HTL blocking solution in prewarmed NbActiv4.

2. Aspirate most of the NbActiv4 from the dish (save a small volume of media to cover cells on the glass coverslip) and replace with 2 mL of JF_646_-HTL blocking solution.

3. Return the dish to the incubator for 1 h.

4. Aspirate blocking solution and replace with fresh, prewarmed NbActiv4. Repeat this wash three times to completely remove unbound dye and CHX.

5. Return the dish to the incubator for 3 h to allow protein synthesis to recover.

*Note: Vary the duration of this recovery step to tune the labeling density of GluA1-HT by JF_549_-HTL. For example, allowing synthesis a greater time to recover will result in higher labeling density.*

6. Prepare 2 mL of JF_549_-HTL labeling solution in prewarmed NbActiv4.

7. Aspirate NbActiv4 and replace with 2 mL of JF_549_-HTL labeling solution.

8. Return neurons to the incubator for 30 min.

9. Aspirate labeling solution and replace with 2 mL of prewarmed imaging buffer. Repeat this wash three times to completely remove unbound dye.

10. Return the dish to the incubator for 30 min.

11. Wash cells one more time with fresh, prewarmed imaging buffer.

**G. Imaging single GluA1-HaloTag-JF_549_ particles**

*Note: The following steps describe imaging particles using highly inclined and laminated optical sheet (HILO) microscopy [24]. Other types of microscopy may also be used, but should be optimized accordingly (see* ***General note #7***).

1. Place MatTek dish with labeled cells onto the microscope stage.

*Note: Maintain a constant temperature of 37 °C during imaging using an environmental control chamber, as changes to temperature can alter the motion of particles.*

2. Using a 640 nm laser and filters appropriate for Cy5 (Cy5 channel), identify a healthy neuron that expresses GluA1-HT labeled with JF_646_-HTL (GluA1-HT-JF_646_^+^). GluA1-HT-JF_646_^+^ neurons will contain a strong signal in the dendrites, especially in the dendritic spines where GluA1 accumulates (**Figure 1D**).

*Note: Although edited neurons can be identified at 100× magnification, it may be easier to first identify a neuron of interest at 20× magnification and then change the magnification to 100×.*

3. Move the field of view to a healthy, spiny secondary dendrite on the GluA1-HT-JF_646_^+^ neuron (**Figure 2B, block**).

4. Adjust the Z position to approximately find the center of the dendrite.

5. View this field of view with a 561 nm laser and filters appropriate for TRITC (TRITC channel). GluA1-HT-JF_549_ should be sparsely labeled, and single particles readily distinguishable (**Figure 2B, chase**).

6. If HILO is used, use the TRITC channel to find the incident angle and illumination direction that produces the best signal-to-noise ratio for single GluA1-HT-JF_549_ particles.

7. Switch back to the Cy5 channel and find another healthy, spiny dendrite on the same cell. Again, adjust the Z position to find the center of the dendrite.

8. Briefly switch to the TRITC channel to view single particles. The incident angle and direction may need minor adjustments to find the best signal-to-noise for GluA1-HT-JF_549_ particles. Make these adjustments quickly to avoid bleaching the single GluA1-HT-JF_549_ particles.

9. Once satisfied, start timelapse imaging at 20–50 ms exposure times.

*Note: We use a multi-camera setup to simultaneously image in the Cy5 and TRITC channels (Figure 2B; Video 1). If a multi-camera setup is not available, record a reference image in the Cy5 channel before starting the timelapse in the TRITC channel. The cameras used in our setup have a field of view of 512 × 512 pixels, with a pixel size of 160 nm. We image the center Z plane for 400–1,000 frames. However, imaging setups with finer Z sectioning may require a Z stack.*

**Video 1.**
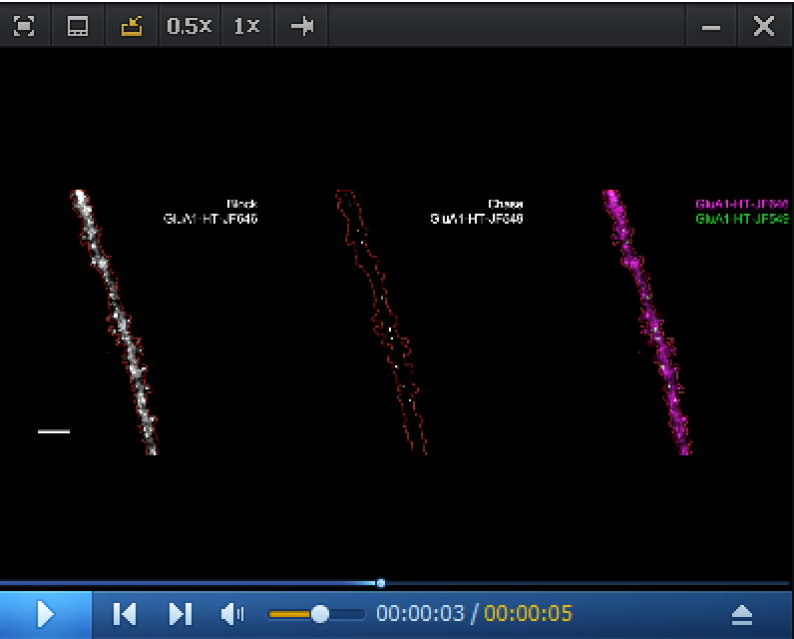
Timelapse sequence of GluA1-HT-JF_549_ after block-and-chase labeling with JF_646_-HTL and JF_549_-HTL in the dendrite of a cultured rat hippocampal neuron. Scale bar, 10 μm. Left: Existing GluA1-HT labeled with JF_646_-HTL (block; GluA1-HT-JF_646_). Center: Newly synthesized GluA1-HT labeled with JF_549_-HTL (chase; GluA1-HT-JF_549_). Right: GluA1-HT-JF_646_ and GluA1-HT-JF_549_ images merged.

**H. Tracking and characterizing single GluA1-HaloTag-JF_549_ particles**

**Figure 3.**
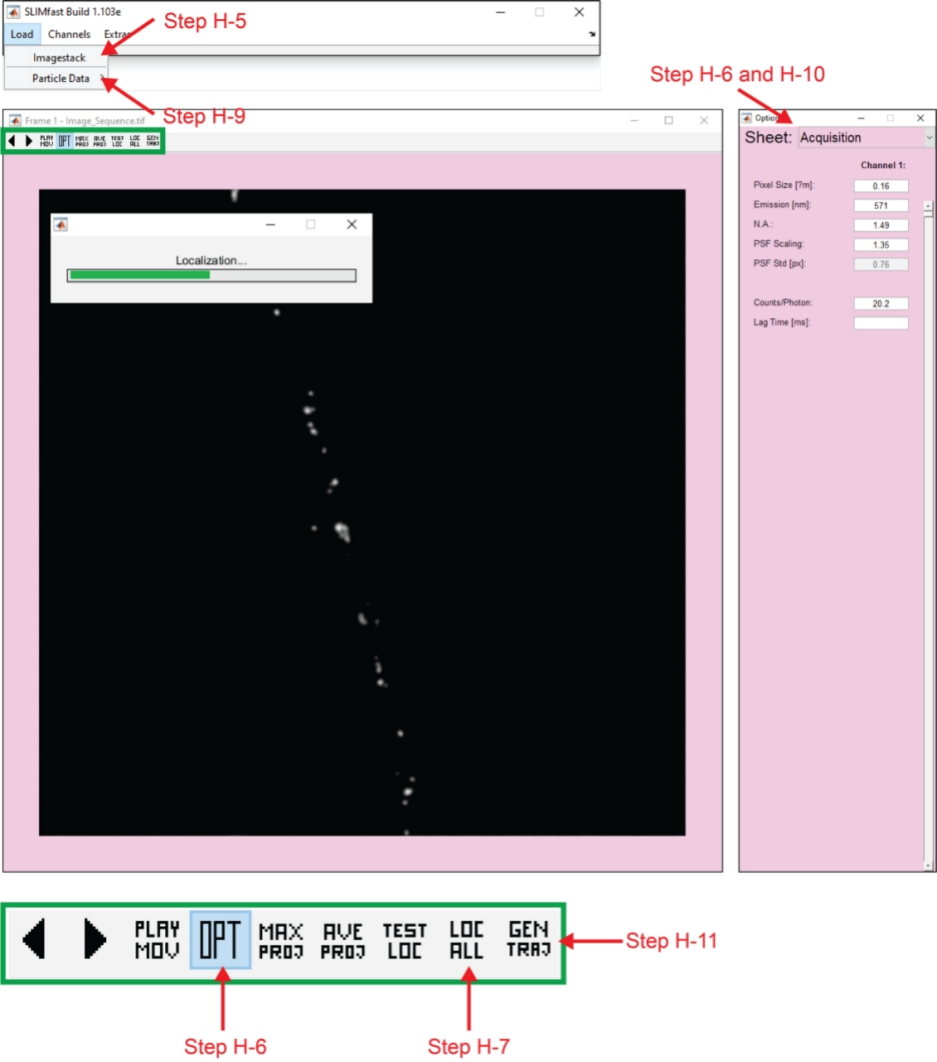
MATLAB graphical user interface for the SLIMfast program. SLIMfast is an in-house MATLAB program based on the multiple-target tracing (MTT) algorithm developed by Serge et al. [20] for single-particle tracking. *Arrows*, cycle through the frames of the timelapse sequence; *PLAY MOV*, play the timelapse sequence; *OPT*, opens a graphical user interface (GUI) with options for acquisition, localization, and tracking parameters; *MAX PROJ*, creates a figure with a maximum intensity projection of the timelapse sequence; *AVE PROJ*, creates a figure with an average intensity projection of the timelapse sequence; *TEST LOC*, locates the particles on the current frame of the timelapse sequence; *LOC ALL*, performs localization on all particles in the timelapse sequence; *GEN TRAJ*, performs reconnection to generate trajectories.

1. Convert image sequences to TIFFs with NIS-Elements or ImageJ.

2. Download SLIMfast, DiffusionSingle, TrackValidator, and HMM_Batch programs for MATLAB.

3. Open MATLAB and create paths to the folder containing the image sequence to be analyzed (e.g., *Image_Sequence.tif*) and folders containing the SLIMfast, DiffusionSingle, TrackValidator, and HMM_Batch MATLAB programs.

4. Use the command *run SLIMfast* in the MATLAB command window, which will open a graphical user interface (GUI) for SLIMfast (Figure 3), a program that uses a multiple-target tracing (MTT) algorithm to achieve 2D single-molecule localization (x,y) [20].

5. Using the SLIMfast GUI, open the image sequence to be analyzed: *Load* → *Imagestack* → *Image_Sequence.tif*. The GUI will display the first image (*Frame 1*) in the image sequence. Icons with commands will appear above the *Frame 1* image (icon functions described in Figure 3).

6. Click on the *OPT* icon, which will open a GUI containing options to modify the imaging conditions and tracking parameters. You can access these parameters using the drop-down menu. First, enter the correct acquisition (*Acquisition*) and localization (*Localization*) parameters, and then close the *OPT* GUI.

*Note: See Table 1 for acquisition and localization parameters that should be changed or optimized according to experimental conditions.*

7. Press the *LOC ALL* icon to execute particle localization. After pressing the *LOC ALL* icon, select a folder to save the files containing the localization data. A progress bar will appear.

8. Once localization is complete, the progress bar will disappear. Close the *Frame 1* GUI.

9. Use the drop-down menu to load the particle data: *Load* → *Particle Data* → *SLIMfast* → *Image_Sequence.rpt*. A maximum intensity projection (MIP) of the image sequence will automatically appear.

10. Press the *OPT* icon. Change tracking parameters (*Tracking)* using the drop-down menu. Optimizing these parameters may be necessary to find the most accurate trajectories. Close the *OPT* GUI once complete.

*Notes:*

*1. Although the default values for most tracking parameters are generally robust and have been previously described in detail [20, 25–28], two critical parameters should be optimized: 1) the maximum expected diffusion coefficient and 2) the maximum OFF-time. The maximum expected diffusion coefficient constrains the step size of particles based on how quickly a particle is predicted to diffuse. The maximum OFF-time determines the number of frames before a trajectory is terminated if the trajectory fails to reconnect (i.e., determines the tolerance for when a particle disappears, either through blinking, bleaching, or because it has moved in Z).*

*2. See Table 1 for critical tracking parameters that may require optimization.*

**Figure 4.**
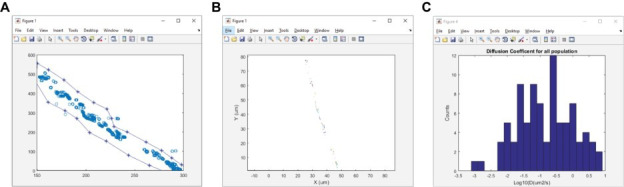
Example figure outputs for the DiffusionSingle program. DiffusionSingle is an in-house MATLAB program that utilizes MSDanalyzer (created by Tarantino et al. [29]) to calculate diffusion coefficients. (A) Overlay of all particles localized in the timelapse sequence. (B) Trajectories reconstructed from the timelapse sequence. (C) Distribution of diffusion coefficients (Log_10_D).

11. Press the *GEN TRAJ* icon to generate trajectories. A progress bar will appear. After the progress bar disappears, save files containing the tracking parameters. Close the SLIMfast GUIs.

12. Return to the MATLAB command window and use the command *run DiffusionSingle*. Open Image_Sequence.rpt_tracked_table.txt.

13. A MATLAB figure will appear, containing particle positions over time (Figure 4A). Use the cursor to outline the region of interest. *DiffusionSingle* will then use MSDanalyzer [29] to compute the diffusion coefficients for each trajectory and produce two figures: 1) the trajectories for particles and 2) a distribution of diffusion coefficients (Figure 4B, C).

*Notes:*

*1. Access the DiffusionSingle.m MATLAB file to change the time between images in the timelapse (i.e., the lag time). We use a lag time of 20–50 ms.*

*2. The fraction of the MSD plot used for fitting to determine the diffusion coefficient can be modified inside the DiffusionSingle.m file.*

*3. Raw data is accessible in the ma_all MAT file in MATLAB.*

**Figure 5.**
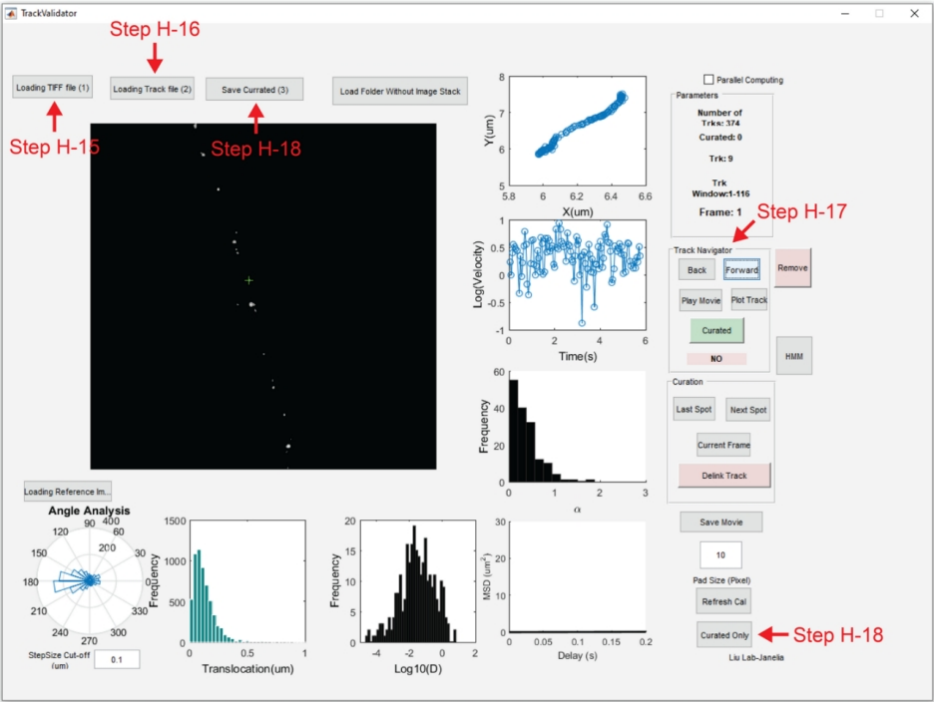
MATLAB graphical user interface for the TrackValidator program. TrackValidator is an in-house MATLAB program for the visualization and curation of trajectories. Using TrackValidator, the motion state of the current trajectory can be determined by pressing the HMM-Bayes analysis icon (HMM) [21]. Alternatively, curated trajectories can be analyzed in batch using the HMM_Batch program.

**Figure 6.**
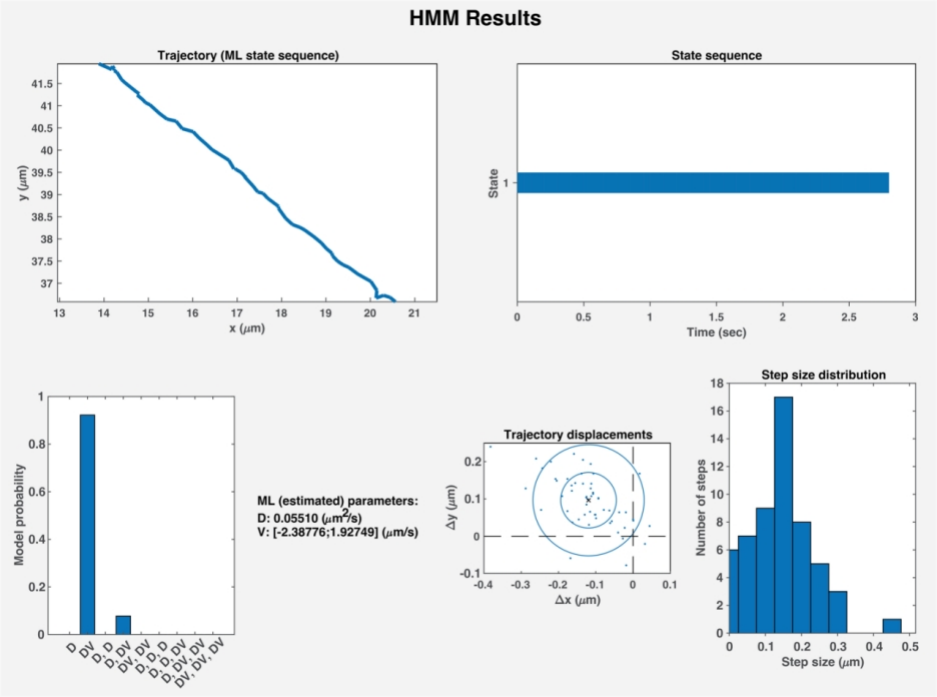
Results generated by HMM-Bayes analysis. HMM-Bayes [21] will generate a figure for each curated trajectory containing 1) the relative xy positions of the particles in a trajectory, 2) the motion state sequence of the trajectory, 3) the probability that the motion state is correctly predicted, 4) the motion parameters, 5) the trajectory displacements, and 6) a step-size distribution between particles. D, diffusion; DV, active transport.

14. Return to the MATLAB command window and use the command *run TrackValidator* to open the TrackValidator GUI (Figure 5).

15. Upload image sequence: *Loading TIFF file (1)* → *Image_Sequence.tif.*

16. Upload track file: *Loading Track file (2)* → *Image_Sequence.rpt_tracked_table.*

17. Using the *Track Navigator* icons, inspect each track for accuracy. To examine whether the localization of a particle is correct for every frame, use the *Last Spot* and *Next Spot* icons. Use the *Delink Track* icon to split a trajectory into two trajectories before and after the current frame. This tool is useful when only part of the trajectory is accurate. If a track is accurate, press the *Curated* icon.

18. Once accurate tracks have been curated, press the *Save Curated (3)* icon and save the MAT file. Move this MAT file into a new folder titled *Batch*. The curated tracks can be reopened using TrackValidator by pressing the *Curated Only* icon and loading the MAT file containing curated tracks.

19. To determine the motion states and parameters using HMM-Bayes [21], use the command *run HMM_Batch* in the MATLAB command window. When prompted, select the *Batch* folder containing the MAT file with curated tracks. *HMM_Batch* will produce and save MATLAB figures containing the motion parameters, state sequences, step-size distributions, and the probability of each motion state for each trajectory (Figure 6).

*Note: Access the HMM_Batch.m MATLAB file to change the lag time.*

**Table 1.** Important parameters to be optimized for single-particle tracking

Acquisition		
**Parameter**	**Description**	**Values in [23]**
Pixel size (μm)	Pixel size used by the camera.	0.16 μm
Emission (nm)	Emission wavelength of the imaged dye. We used JF_549 _for the chase.	571 nm
Numerical aperture (N.A.)	The numerical aperture of the used objective. We use a 100× Apo TIRF objective.	1.49
Counts/photon	Adjust according to camera specifications.	20.2
Lag time (ms)	Time interval between images.	50 ms
**Localization**		
**Parameter**	**Description**	**Values in [23]**
Deflation loops	Determines how often the detection process will be iterated. After each iteration, detected particles, represented by their Gaussian intensity profile, will be removed from the image. This allows low-intensity particles to be detected.	3
**Tracking**		
**Parameter**	**Description**	**Values in [23]**
Maximum expected diffusion coefficient (μm^2^/s)	Constrains the step size of particles based on how quickly a particle is predicted to diffuse.	1 μm^2^/s
Maximum number of competitors	Determines the maximum number of trajectories that can compete for a particle.	3
Maximum OFF-time	The number of frames before a trajectory is terminated if the trajectory fails to reconnect.	1

**I. GluA1-HaloTag-JF_549_ vesicle identification**

1. Curate trajectories that exhibit active transport (i.e., trajectories predicted by HMM-Bayes to exhibit active transport, DV, with high probability; see **Figure 6**).

2. Determine the bleaching rate for GluA1-HT-JF_549_ particles to separate single receptors from vesicles.

a. Plot the fluorescence intensity over time for a GluA1-HT-JF_549_ particle using NIS-Elements or ImageJ. JF_549_ will bleach in a stepwise fashion.

b. Determine the number of frames between each bleaching step.

c. Repeat this analysis for several GluA1-HT-JF_549_ particles located at different positions in the dendrite.

d. Determine the bleaching step that required the most frames and use this as a threshold for the number of frames required to bleach a single JF_549_ particle.

e. Multiply this number of frames by 4. This is the maximum number of frames required for a GluA1-HT homomeric receptor (i.e., four GluA1 subunits) to be bleached. Filter out trajectories (that exhibit only diffusion, not active transport) that terminate in fewer frames. Under the imaging conditions we previously used, we found that single GluA1-HT-JF_549_ homomers bleach in fewer than 100 frames. This analysis should be performed as imaging conditions change.

3. For trajectories with diffusion, filter out particles that have a diffusion coefficient greater than 0.45 μm^2^/s and less than 0.02 μm^2^/s. We previously found that particles with very high and low diffusion rates are likely to be single receptors diffusing on the neuronal surface or clusters of receptors trapped inside postsynaptic densities [23].

## Validation of protocol

This protocol or parts of it have been used and validated in the following research articles:

- Suzuki et al. [17]. In vivo genome editing via CRISPR/Cas9 mediated homology-independent targeted integration. *Nature* (Figure 2; Extended Data Figure 5; Extended Data Figure 7).
  This research article describes the development and validation of homology-independent targeted integration as a tool to edit genes in post-mitotic cells, including cultured neurons.
- Grimm et al. [19]. A general method to improve fluorophores for live-cell and single-molecule microscopy. *Nature methods.*
  This research article describes the development of JF_646_ and JF_549_ and their utility in cellular imaging.
- Wong et al. [23]. Plasticity-induced actin polymerization in the dendritic shaft regulates intracellular AMPA receptor trafficking. *eLife* (Figure 1A–D; Figure 1: Figure Supplement 1–8).
  This research article describes the validation of the GluA1-HT knock-in and also describes the use of single-particle tracking to characterize the motion of GluA1-HT^+^ vesicles. This includes characterizing the knock-in efficiency of HaloTag into GluA1 and the rate of errors; demonstrating that GluA1-HT functions and is trafficked in a manner similar to untagged GluA1; and characterizing and validating the motion of GluA1-HT^+^ vesicles.
- Sergé et al. [20]. Robust single-particle tracking in live-cell time-lapse sequences. *Nature methods.*
  This research article describes multiple-target tracing (MTT) and its utility in single-particle tracking.
- Monnier et al. [21]. Inferring transient particle transport dynamics in live cells. *Nature methods.*
  This research article describes the development and validation of HMM-Bayes as a tool to characterize the motion states of single particles.
- Liu et al. [27]. Direct Observation of Compartment-Specific Localization and Dynamics of Voltage-Gated Sodium Channels. *J Neurosci* (Figure 13).
  This research article uses both the block-and-chase protocol and MTT to track vesicles containing voltage-gated sodium channels.
- Liu et al. [28]. Visualizing long-term single-molecule dynamics in vivo by stochastic protein labeling. *Proc Natl Acad Sci USA* (Figures 2 and 3; Figure S14; Table S1).
  This research article uses MTT and HMM-Bayes to track and characterize the motion of synaptic vesicles.

## General notes and troubleshooting

**General notes**

1. Sterile technique should be used throughout this protocol to prevent contamination. Use barrier tips for pipetting. Perform all steps in a clean biosafety cabinet. Ensure the incubator is clean and free of contaminants.

2. Prewarm media and buffers that contact cultured neurons to 37 °C unless stated otherwise.

3. We previously studied AMPAR trafficking during plasticity in the hippocampus [23], but these techniques are also applicable to cortical cells.

4. We have not tested cationic lipid-based delivery of the HITI plasmids. Because gene editing rates are low, we try to achieve the highest transfection/transduction rate possible. In our hands, AAV infection has the highest plasmid delivery rate once the titer has been optimized (~90% of neurons are transduced). We find nucleofection also has a higher transfection efficiency than chemical transfection in neurons (50%–70% cells transfected vs. ~5% cells transfected, respectively). Nevertheless, lipid-based transfection is less toxic than nucleofection, and thus may be a viable alternative to nucleofection once optimized.

5. We performed the block-and-chase protocol with JF_646_-HTL and JF_549_-HTL primarily because of the brightness and stability of these dyes, and because of their appropriate cell permeability and binding kinetics. Nevertheless, other dyes with different properties (e.g., emission wavelengths) may also be used if they are conjugated to HaloTag ligand. We previously performed experiments with the cell-membrane impermeable version of JF_549_-HTL (JF_549_i-HTL) to study the surface trafficking of GluA1-HT [23].

6. This protocol can be used to achieve sparse labeling for proteins other than AMPARs, but key considerations should be made first. Most critically, an appropriate location for the insertion of HaloTag should be identified, and the expression, localization, and function of the tagged protein should be validated [23]. For example, this protocol has also been used to probe the transport of voltage-gated sodium channels [28].

7. In our publication [23], we used highly inclined and laminated optical sheet (HILO) microscopy to improve the signal-to-noise ratio of single particles [24]. Single particles can also be imaged using standard widefield microscopy and spinning disk confocal microscopy, but important considerations should be made to improve the signal-to-noise ratio and decrease bleaching. For example, a sufficiently powerful light source (ideally a laser) should be used for excitation, and a sensitive and fast camera should be used for acquisition. For widefield microscopes, the labeling density may need to be further optimized (see **Troubleshooting #7**). For spinning disk confocal microscopy, dendrites should be flat to minimize the loss of particles from the field of view as they move up and down in Z. An objective with a lower numerical aperture may be used at the cost of some resolution. Alternatively, it may also be possible to take a small Z stack for every time point, depending on the stage speed and exposure time. Regardless of the setup, the workflow should remain the same: 1) identify a dendrite using the GluA1-HT-JF_646_ labeling; 2) optimize the signal-to-noise ratio and bleaching rate of GluA1-HT-JF_549_ particles; 3) find another healthy dendrite using the GluA1-HT-JF_646_ labeling; and 4) commence timelapse of GluA1-HT-JF_549_ particles.

8. Stimulation can be performed after labeling and before (or during) imaging. For example, we tracked GluA1-HT^+^ vesicles after glycine-induced chemical long-term potentiation [23].

9. We used MATLAB version 2015B for Windows OS for single-particle tracking and HMM-Bayes analysis, but programs should be compatible with other versions of MATLAB for Windows OS.

10. HMM-Bayes analysis can also be used to characterize particles with multiple motion states and predict the probability that a particle will switch between these motion states. This is a useful tool to describe vesicle motion as vesicles frequently transition between active transport and diffusion (i.e., pausing).

11. This single-particle tracking pipeline was created in-house at the Janelia Research Campus. However, it is based on theories and algorithms described elsewhere and generously disseminated by their original authors. SLIMfast is based on the multiple-target tracing (MTT) algorithm for SPT [20,25]. DiffusionSingle integrates MSDanalyzer [29] to measure diffusion coefficients. The TrackValidator program was created by Zhe J. Liu at the Janelia Research Campus. HMM-Bayes was developed by Monnier et al. [21].

12. Other particle tracking strategies may also be applied during image analysis. Kymograph analysis can be used when studying the active transport of vesicles.

**Troubleshooting**

Problem 1: Cultured neurons adhere poorly to the coverslip, and cells grow into bundles.

Possible cause: PDL coating is insufficient.

Solution: Make sure PDL stock is completely dissolved before making aliquots. Thaw a fresh PDL stock and use the diluted working concentration of PDL immediately. Do not repeatedly freeze and thaw PDL stocks. Ensure that the PDL covers the glass coverslip completely. Incubate the PDL on the MatTek dish for at least 2 h. Using freshly coated dishes is recommended, but PDL-coated dishes may be used up to 2 weeks after coating if they are properly stored in a sterile environment.

Problem 2: Cultured neurons are unhealthy or die several days after plating (without nucleofection).

Possible cause: NbActiv4 is expired, contaminated, or comes from a bad lot.

Solution: Ensure that NbActiv4 is not expired or contaminated. Do not use an opened bottle of NbActiv4 if it is over one month old. If the bottle is neither expired nor contaminated, it may come from a bad lot. In this case, contact the manufacturer and request a bottle from a different lot.

Problem 3: Cultured neurons are unhealthy or die after nucleofection.

Possible cause: Neuronal health is sensitive to P3 Nucleofector^®^ solution and nucleofection.

Solution 1: Use AAV if available.

Solution 2: Handle neurons carefully but quickly during nucleofection. After neurons have been resuspended in P3 Nucleofector^®^ solution, perform the nucleofection and add plating media to the cells as soon as possible to allow the cells to recover. Pipette neurons slowly and carefully. Minimize the number of times the neurons are pipetted up and down, especially after nucleofection.

Problem 4: Few or no HaloTag^+^ cells after transfection/transduction.

Possible cause 1: If AAV was used to deliver the plasmids, then the viruses may have gone bad, or an insufficient copy number of viruses was used.

Solution 1: Use a new batch of AAV. Avoid repeatedly freezing and thawing virus. Increase the genomic copies of each virus. Generally, AAVs are well tolerated by cultured rat neurons. Nevertheless, we found diminishing returns in knock-in efficiency beyond 1 × 10^11^ genomic copies of each virus. Test whether nucleofection results in a higher number of HaloTag^+^ cells to determine whether the problem lies with the virus.

Possible cause 2: If nucleofection was used to deliver the plasmids, there may be a problem with the plasmid DNA or nucleofection.

Solution 2: Ensure the plasmids are dissolved in endotoxin-free H_2_O (endotoxin decreases transfection efficiency). Discard complete P3 Nucleofector^®^ solution (P3 Nucleofector^®^ solution plus supplement 1) if it was mixed over 6 months prior to use. Ensure there were no errors during nucleofection (an error notice will appear on the 4D-Nucleofector^®^ unit). Co-transfect neurons with another plasmid that expresses a fluorescent marker driven by a neuron-specific promoter (e.g., GFP driven by the synapsin promoter) to determine whether issues arise during nucleofection. The transfection marker can also be used to ensure that HaloTag is being expressed in neurons and to estimate the transfection efficiency.

Possible cause 3: Janelia Fluor dyes have gone bad.

Solution 3: Use freshly dissolved dyes. Avoid repeatedly freezing and thawing dyes. Store at -20 °C long term. Avoid exposure to light.

Problem 5: Nonspecific JF_646_-HTL or JF_549_-HTL labeling.

Possible cause: HTL dye concentration is too high, duration of labeling is too long, or washes are insufficient.

Solution: Use lower concentrations of JF_646_-HTL and JF_549_-HTL. 5–10 nM of dye is sufficient to label HaloTag^+^ neurons. Reduce labeling duration. 15 min of JF_549_-HTL labeling is sufficient. Increase the number of washes.

Problem 6: HaloTag^+^ cells are difficult to identify because they have a weak signal after JF_646_-HTL or JF_549_-HTL labeling.

Possible cause: Low GluA1 expression.

Solution: Use a strong excitation light source and a sensitive camera. The native promoter of *Gria1* will express less GluA1 than an overexpression plasmid. Relatively low expression is an inherent feature of editing the genomic copy of *Gria1* that makes sparse labeling possible.

Problem 7: Labeling density of GluA1-HT-JF_549_ particles is too high or too low.

Possible cause: Recovery time for GluA1-HT synthesis after removing CHX and JF_646_-HTL is inappropriate.

Solution 1: Vary the recovery time after washing out CHX and JF_646_-HTL. Increasing recovery time will allow for the synthesis of more GluA1-HT, leading to increased particle densities. Decreasing the recovery time will result in lower particle densities. The JF_549_-HTL labeling duration may also be increased, but incubating the neurons with JF dye for too long will increase nonspecific labeling.

Solution 2: Find a neuron that has the appropriate labeling density. The rate of GluA1-HT expression is dependent on cell state. Consequently, labeling densities will vary from cell to cell in the same plate.

Problem 8: Neurons appear unhealthy after labeling.

Possible cause: Full volume of NbActiv4 or imaging buffer removed during wash or labeling steps.

Solution: Leave a small volume of NbActiv4 or imaging buffer covering the cells during wash and labeling steps. Completely removing the NbActiv4 or imaging buffer can lead to dendritic beading.

Problem 9: Particles bleach very quickly or have a low signal-to-noise ratio.

Possible cause: Imaging setup/parameters are inappropriate.

Solution: Optimize the laser power and exposure times to balance signal-to-noise and bleaching rate. Use the lowest laser power possible where single particles are still clearly detectable. A shorter exposure time is also preferred as more information can be collected during a timelapse. HILO imaging can further improve the signal-to-noise ratio, but the incident angle and laser direction need to be adjusted accordingly.

Problem 10: GluA1-HT-JF_549_ particles are not accurately tracked.

Possible cause: Acquisition, localization, and tracking parameters are inappropriate.

Solution: First, ensure the acquisition parameters in SLIMfast.m are correct. Next, optimize the maximum expected diffusion coefficient and the maximum OFF-time. Another parameter that may be optimized is the maximum number of competitors. This value determines the maximum number of trajectories that can compete for a particle.
